# Epilepsy in Children: From Diagnosis to Treatment with Focus on Emergency

**DOI:** 10.3390/jcm8010039

**Published:** 2019-01-02

**Authors:** Carmelo Minardi, Roberta Minacapelli, Pietro Valastro, Francesco Vasile, Sofia Pitino, Piero Pavone, Marinella Astuto, Paolo Murabito

**Affiliations:** 1 Department of Anesthesiology, AOU Policlinico—Vittorio Emanuele, University of Catania Via S. Sofia, 78, 95123 Catania, Italy; minardi.carmelo@virgilio.it (C.M.); robertaminacapelli@gmail.com (R.M.); valastrop87@gmail.com (P.V.); Frankie.Vas@hotmail.it (F.V.); sofp85@gmail.com (S.P.); astmar@tiscali.it (M.A.); paolomurabito@tiscali.it (P.M.); 2 Department of Pediatrics, AOU Policlinico—Vittorio Emanuele, University of Catania Via S. Sofia, 78, 95123 Catania, Italy; ppavone@unict.it

**Keywords:** seizures, status epilepticus, children

## Abstract

Seizures are defined as a transient occurrence of signs and symptoms due to the abnormal, excessive, or synchronous neuronal activity in the brain characterized by abrupt and involuntary skeletal muscle activity. An early diagnosis, treatment, and specific medical support must be performed to prevent Status Epilepticus (SE). Seizure onset, especially in the child population, is related to specific risk factors like positive family history, fever, infections, neurological comorbidity, premature birth, mother’s alcohol abuse, and smoking in pregnancy. Early death risk in children without neurological comorbidity is similar to the general population. Diagnosis is generally based on the identification of continuous or recurrent seizures but Electroencephalogram (EEG) evaluation could be useful if SE condition is suspected. The main goal of therapy is to counteract the pathological mechanism which occurs in SE before neural cells are irreversibly damaged. According to the latest International Guidelines and Recommendations of seizure related diseases, a schematic and multi-stage pharmacological and diagnostic approach is proposed especially in the management of SE and its related causes in children. First measures should focus on early and appropriate drugs administration at adequate dosage, airway management, monitoring vital signs, Pediatric Intensive Care Unit (PICU) admission, and management of parent anxiety.

## 1. Introduction

The emergency department generally is the place where children affected by seizures receive first treatment and medical support. Proper skills of physicians are essential for early diagnosis, treatment, and adequate communication with the parents.

Seizures are defined as a transient occurrence of signs and symptoms due to the abnormal, excessive, or synchronous neuronal activity in the brain characterized by abrupt and involuntary skeletal muscles activity. The adjective “transient” in the definition, indicates a time frame with a clear onset and remission [1]. Status epilepticus (SE) is a condition resulting either from the failure of the mechanisms responsible for seizure termination or from the initiation of a mechanism, which leads to abnormally, prolonged seizures (for a time period of 5 min or more). It is a condition, which can have long-term consequences (especially if its duration is more than 30 min) including neuronal death, neuronal injury, and alteration of neuronal network, depending on the type and duration of seizures [1]. Febrile seizures are defined as critical seizures which occurs in children aged between 1 month and 6 years with temperature rise over 38 °C and without signs of infectious disease of the central nervous system (CNS) [2].

In 2014 the International League Against Epilepsy (ILAE) Task Force proposed the operational (practical) clinical definition of epilepsy, intended as a disease of the brain defined by any of the following conditions:

1. At least two unprovoked (or reflex) seizures occurring >24 h apart 
2. One unprovoked (or reflex) seizure and a probability of further seizures similar to the general recurrence risk (at least 60%) after two unprovoked seizures, occurring over the next 10 years
3. Diagnosis of an epilepsy syndrome

Epilepsy is considered to be resolved for individuals who had an age-dependent epilepsy syndrome but are now past the applicable age or those who have remained seizure-free for the last 10 years, with no seizure medicines for the last 5 years [3].

The incidence of epilepsy varies between industrialized countries and developing ones. In Western countries, new cases per year are estimated to be 33.3–82/100,000, [4] in contrast to the maximum incidence of 187/100,000 estimated in developing countries [4,5]. In particular, recent studies showed that the maximum incidence occurs in the first year of age with a rate of 102/100,000 cases per year, just like the age range from 1 to 12 [4]; in children from 11 to 17 years old incidence is 21–24/100,000 cases [4,5]. Previous studies suggest that the total incidence of epilepsy is constant from 25 years, showing a slight increase in males [4].

In Italy, epilepsy incidence is 48.35/100,000 new cases per year and it is comparable with data recorded in the other industrialized countries. The peak of incidence occurs in children younger than 15 years old (50.14/100,000 new cases per year) and especially in the first year of life with an incidence of 92.8/100,000 new cases per year. In this regard, it should be taken into due account that the child’s immature CNS is more susceptible to seizures and at the same time refractory to the consequences of an acute attack. Finally, incidence is higher in males than in females [6].

From 2015 to 2017 the ILAE Task force revised concepts, definition, and classification of seizures, epilepsy, and Status Epilepticus. In the classification of seizures (Table 1) levels can be skipped.

Moreover, the diagnosis of epilepsy has become a multilevel process, which is designed to allow the classification of epilepsy in different clinical environments, meaning that different levels of classification will be possible depending on the available resources. After the presentation of seizures in a patient, the clinician makes a diagnosis working through several critical steps, excluding, however, any other possible causes for the clinical condition (epilepsy-imitators [8]). Indeed, the classification includes three levels: seizure types, epilepsy type, epilepsy syndrome (Table 2). Where possible, a diagnosis at all three levels should be sought as well as the etiology of the individual’s epilepsy [9].

On SE, the most common causes in children are fever and infections of the CNS. Other causes include hyponatremia, accidental ingestion of toxic agents, abnormalities of the CNS, genetic and metabolic disorders (phenylketonuria, hypocalcemia, hypoglycemia, hypomagnesemia).

The pathophysiological course of SE in children depends on the absence of anatomical abnormalities and pre-existing predisposing conditions of CNS.

SE is a condition resulting either from the failure of the mechanisms responsible for seizure termination or from the initiation of mechanisms which lead to abnormally prolonged seizures (after time point t_1_). It is a condition that can have long-term consequences (after time point t_2_), including neuronal death, neuronal injury, and alteration of neuronal networks, depending on the type and duration of seizures [1].

This definition is conceptual, with two operational dimensions: the first is the length of the seizure and the time point (t_1_) at which the seizure should be regarded as an “abnormally prolonged seizure.” The second time point (t_2_) is the time of ongoing seizure activity beyond which there is a risk of long-term consequences.

### 1.1. Classification of SE

Status Epilepticus is classified according to the International League Against Epilepsy (ILAE) guidelines [1] into four categories: semiologic (Table 3), etiologic (Table 4), EEG pattern (Table 5), age-related (Table 6).

### 1.2. Risk Factors

The principal risk factors for seizures in children are correlated with: positive family history [10], high temperature [11], mental disability [12], delayed discharge from NICU or premature birth [10], mother’s alcohol abuse and smoking in pregnancy doubles the risk of seizure incidence [13]. Moreover in 30% of children in which the first episode of seizures occurs, the probability of recurrent episodes is increased.

Instead risks factors of recurrent febrile seizures include: small age and duration of first episode of seizures, low temperature during the first episode, positive familiar history for febrile seizures in a first degree relative, short timeframe from temperature elevation, and seizure onset [10].

Patients with all these risk factors show more than 70% probability of a recurrent episode of seizures; in contrast patients with none of them have a probability of a recurrent episode of seizure lower than 20% [14,15].

### 1.3. Mortality

The mortality rate in people affected by epilepsy is 2–4 times higher than the rest of the population, and 5–10 times higher in children.

Early death risk in children without neurological comorbidity is similar to the general population and lots of deaths are not related to seizures themselves but to the neurological preexisting disability.

This risk increase is a consequence of: lethal neuro-metabolic alterations, systemic complications (consequence of neuro-disability), death directly related to seizures.

This group includes sudden unexpected death in epilepsy (SUDEP), that represents the most common cause of death related to epilepsy in children: it is uncommon but death risk increases if epilepsy persists until the young-adult age [12,13].

Other causes of death could be: seizure related (ab-ingestis), natural causes related (brain tumors), non-natural causes (suicide or accidental death).

Global mortality rates are between 2.7 and 6.9 death per 1000 children every year; SUDEP related mortality in children is about 1.1–2 cases/10,000 children per year [13].

### 1.4. Pathophysiology

The exact mechanism of seizure onset is unknown. There could be either a deficit of neuronal inhibition or an excess of excitatory stimuli. Most authors suggest that the onset of seizures depends on a deficit in the neuronal inhibition, in particular γ-Aminobutyric acid (GABA) deficit [16], the most important neurotransmitter of CNS; alternatively it depends on the alteration of the GABA function which determines a prolonged and high intensity stimulation.

Other studies, in experimental animal models, demonstrated that N-methyl-D-aspartate (NMDA) and alpha-amino-3-hydroxy-5-methyl-4-isoxazole-propionic acid, both glutamate receptors, the most important excitatory receptor of CNS, are involved in seizure physiopathology [16]. Febrile seizures occur in young children whose convulsive threshold is lower.

Children are more exposed to frequent infections like: respiratory high tract infections, otitis media, viral infection where children present high temperature [17,18]. Animal models suggest the central role of inflammatory mediators like IL-1 that could cause an increase in neuronal stimulation and the onset of febrile seizures [17].

Preliminary studies in children seem to confirm this hypothesis but its clinical and pathological meaning is still unknown. Febrile seizures could underline a severe pathological process like meningitis, encephalitis, and cerebral abscess [17].

Viral infections seem to be involved in the pathogenesis of seizures. Recent studies show that HHSV-6 (Human herpes simplex virus-6) and Rubivirus could be found in 20% of patients affected by febrile seizures for the first time [18,19]. Finally, other reports also suggest that Shigella related gastroenteritis has been associated with febrile seizures [20].

## 2. Diagnosis

The most challenging condition, which happens to be treated during an emergency, is the status epilepticus. Because of this, diagnosis and treatment sections are focused on this clinical state. 

Clinical presentation in status epilepticus varies. It depends on the type of seizures, stage, and previous state conditions of the pediatric patient. Diagnosis is based on the identification of continuous or recurrent seizures, and it is easy to recognize during the clinical manifestation.

After persisting status epilepticus, despite disappearance of motor manifestations, it is difficult to exclude non-epilepticus continuous status.

A complete instrumental evaluation can be requested in case of first clinical presentation of SE, or in case of complicated SE, comorbidity, and in infants [21].

Literature suggests that in pediatric age routine, serologic examinations are not justified, because of the low frequency of abnormal values. The only abnormal test in more than 20% of patients is hypoglycemia [21].

In patients with status epilepticus and body temperature above 38.5 °C, a lumbar puncture could be considered, when infectious etiology is suspected. Temperature, leukocytosis, and pleocytosis in cerebro-spinal fluid may be present in SE even if infections in the central nervous system are absent.

American Association of Pediatrics (AAP) guidelines in medical management of pediatric patients with febrile seizures do not suggest performing diagnostic tests routinely, including lumbar puncture, except if it is requested by the state of the condition [19].

A lumbar puncture is firmly recommended in all patients under one-year age that present temperature and seizures [14].

American College of Emergency Physician (ACEP) guidelines suggest that the lumbar puncture should be requested in cases of immune-compromission, clinical signs of meningitis, persisting seizures, and recent CNS infections [19].

Computerized Tomography (CT) is requested during the first clinical presentation of seizures and in clinical conditions that could increase the risk of complications.

An encephalic CT without contrast media is the first test recommended to diagnose neoformations, head injury, hemorrhages, and/or cerebral infarcts. A CT with contrast media could be necessary to confirm suspected diagnosis of brain tumors or subdural hematoma.

A study has shown that pediatric patients with complex febrile seizures and normal clinical examination, and pediatric patients with febrile seizures without evident acute cause in anamnesis rarely have a positive CT. So this examination could be postponed [14].

The use of EEG in the emergency room is restricted to differential diagnosis. EEG should be considered every time SE is suspected.

Research of SE causes should proceed in parallel with treatment, and good knowledge is required because optimal treatment includes the prevention of recurrent SE.

## 3. Treatment of SE

The main goal in therapy during SE is to stop seizures before neural cells are irreversibly damaged. SE is difficult to control as the duration increases; for this reason, it is important to start an early target pharmacological treatment. 

The most important thing in pharmacological treatment is rapid implementation of a clear protocol, adjusting doses to the weight of the patient. Therefore, in the case of refractory SE the treatment should be as fast as possible.

The 2017 ILAE recommendations [22] relate pharmacological treatment to time. So three time-points are described here:

- T1 is the period in which the emergency treatment of SE should be started.
- T2 is the period after which seizures could result in neural cell death, modifications in neural networks, and functional deficiency.
- T3 is characterized by refractory SE: SE continues despite the treatment. In this case, hospitalization and PICU admission are recommended.

There is also a period called T4. It is characterized by a super refractory SE, that continues for more than 24 h. In this case, it is necessary to have advanced life support.

### 3.1. General Support Measures

The first approach in SE should focus on airway management and adequate ventilation and circulation. It is important to safeguard patients from injuries caused by uncontrolled movement. It is also important to place the patient in a lateral position to prevent inhalation, and position a peripheral venous catheter.

Monitoring vital signs (heart rate, blood pressure, oxygen saturation, and temperature) is essential to evaluate the course of SE. A rapid blood test should be done to recognize hypoglycemia or poisoning [23].

Most of the drugs used to treat SE suppress respiratory drive. Therefore, it is important to take precautions to recognize and treat their side effects.

### 3.2. Anticonvulsant Drugs in Emergency

Guidelines in the treatment of SE give the basis to manage SE optimally in the emergency room; 80% of patients with simple convulsion respond to initial treatment, including those who will develop an SE.

The most important factor is to use effective drugs at the appropriate dosages. Therapy can be optimized by choosing the correct sequence of drugs (Table 7).

Benzodiazepines are considered the first choice in the initial treatment of seizures and SE in pre-hospital emergency care. They increase inhibition of GABA receptors, have rapid onset and are effective in 79% of patients in SE.

Barbiturates increase inhibition of GABA receptors. Fenobarbital is one of the most commonly used. However, it is difficult to manage because of its long half time.

Phenobarbital and Phenytoin are considered second-class drugs to treat seizures and SE, and they are usually administrated when benzodiazepines fail. Side effects are: sedation, respiratory depression, and hypotension. So airway management and cardiovascular treatment should be considered as priority [24].

Phenobarbital is the antiepileptic drug often used in neonatal seizures, although Phenytoin is equally effective.

Valproic acid is important in refractory SE (stage 2 in 2017 ILAE recommendations) [22].

Propofol is an anesthetic agent with anticonvulsant activity. It is used in refractory SE. The disadvantages are the short half-life and rapid metabolism that can make convulsions worse. The main side effects are respiratory depression and hypotension because of myocardial depression [25,26]. High doses of Propofol in continuous rate infusion should be limited to a short period, generally no more than 24–48 h in order to prevent Propofol infusion syndrome [27].

## 4. Remarks on Convulsions and Pediatric SE

Pediatric patients with head injury and 3–8 Glasgow Coma Scale (GCS) risk developing seizures and it is recommended to prevent them by prophylaxis. Most seizures in pediatric patients and teenagers can be treated by oral valproic acid. In particular, juvenile myoclonic epilepsy (JME) can take advantage of it. Young adults that do not sleep much and drink alcohol can show generalized seizures in the morning [28]. In these patients, valproic acid is a very good drug to use in emergency [29].

## 5. Parents Training for the Future

Parents must be prepared to know what to do if their children show seizures. They should call the emergency number if seizures persist for more than 10 min, and if the post convulsive state lasts longer than 30 min. Moreover, they should be informed about the benign nature of febrile seizures. In fact they are not connected to neurological problems or physically slow development. Parents must pay particular attention to their sons, because studies have proved that febrile seizures are inclined to be recurrent in a family [30].

## 6. Conclusions

Pediatric seizures and SE are emergencies that request early and effective treatment. Everyone is aware that for all this the patients outcome can be improved using antiepileptic drugs at the appropriate dose. Further studies should focus on the management of a pediatric patient’s convulsions or SE through improvement of treatment taking into due account that airway management is priority in pediatric patients with seizures or SE; children with febrile seizures in anamnesis must be evaluated through neurological examination and monitoring of mental development, causes of fever must always be investigated and treated, other causes of seizures must be excluded, and parent anxiety must be controlled.

## Figures and Tables

**Table 1:** Classification of seizure types-expanded version [7].

Seizure Types		Prominent Features		
Focal Onset	Awake/impaired awarness	Motor onset Automatism Atonic Clonic Epileptic spasm Hyperkinetic Myocloniconic Tonic	Non motor onset Autonomic Behavior arrest Cognitive Emotional Sensory	Focal to bilateral tonic clonic
Generalized Onset		Motor Tonic-clonic Tonic Clonic Myoclonic Myoclonic-tonic-clonic Myoclonic-atonic Epileptic spasm	Non motor Typical Atypical Myoclonic Eyelid myoclonia	
Unknown Onset		Motor Tonic-clonic Epileptic spasm	Non motor Behavior arrest	Unclassified

**Table 2:** Classification of the epilepsies [9].

Co-morbidities		
Seizures types Focal Generalized Unknown	Epilepsy types Focal Generalized Combined generalized and focal Unknown	Epilepsy syndromes
Etiology Structural, Genetic, Infectious, Metabolic, Immune, Unknown		

**Table 3:** Semeiologic classification of Status Epilepticus (SE).

Prominent motor symptoms	Convulsive SE	Generalized convulsive	
		Focal onset evolving into bilateral convulsive SE	
		Unknown whether focal or generalized	
	Myoclonic SE	With coma	
		Without coma	
	Focal motor	Repeated focal motor seizures (Jacksonian)	
		Epilepsia partialis continua (EPC)	
		Adversive status	
		Oculoclonic status	
		Ictal paresis	
	Tonic status		
	Hyperkinetic SE		
Without prominent motor symptoms or Non-convulsive status epilepticus (NCSE)	NCSE with coma		
	NCSE without coma	Generalized	Typical absence status
			Atypical absence status
			Myoclonic absence status
		Focal	Without impairment of consciousness
			Aphasic status
			With impairment of consciousness
		Unknown whether focal or generalized	Autonomic SE

**Table 4:** Etiologic classification of SE.

Known	Acute	Stroke, Intoxication, Malaria, Encephalitis, etc.
	Remote	Post traumatic, Post encephalitic, Post stroke, etc.
	Progressive	Brain tumors, Lafora’s disease, Dementias
	SE in defined electro clinical syndromes	
Unknown	Cryptogenetic	

**Table 5:** Electroencephalogram EEG related SE classification.

Location	Generalized
	Lateralized
	Bilateral independent
	Multifocal
Pattern	Periodic discharges
	Number of phases
	Spike-and-wave/sharp-and-wave plus subtypes.
Morphology	Sharpness
	Number of phases
	Absolute and relative amplitude
	Polarity
Time related features	Prevalence
	Frequency
	Duration
	Onset
	Dynamics
Modulation	Stimulus-induced vs. spontaneous
Effect of intervention on EEG	

**Table 6:** Seizure age-related classification.

SE occurring in neonatal and infantile-onset epilepsy syndromes	Tonic status (Ohtahara’s Syndrome, West’s syndrome)
	Myoclonic status in Dravet syndrome
	Focal status
	Febrile SE
SE occurring mainly in childhood and adolescent	Autonomic in early onset benign childhood occipital epilepsy Panayiotopoulos Syndrome)
	NCSE in specific childhood epilepsy syndromes and etiologys (Ring Cromosome 20, Angelman Syndrome)
	Tonic status in Lennox–Gastaut syndrome
	Myoclonic status in progressive myoclonus epilepsies
	Electrical status epilepticus in slow wave sleep (ESES)
	Aphasic status in Landau–Kleffner Syndrome
SE occurring mainly in adolescents and adulthood	Myoclonic status in juvenile myoclonic epilepsy
	Absence status in juvenile myoclonic epilepsy
	Myoclonic status in Down syndrome
SE occurring mainly in the elderly	Myoclonic status in Alzheimer’s disease
	NCSE in Creutzfeldt–Jakob disease
	De novo (or relapsing) absence status of later life

**Table 7:** Pharmacological therapy.

T1		T2		T3	
Early phase Status Epilepticus		Clear Status Epilepticus		Refractory Status Epilepticus	
				Hospitalization in PICU	
***Phase 1*** ***5–10 min***	Lorazepam: 0.1 mg/kg. 4 mg max. If it is necessary, it can be repeated once	***Phase 2*** ***10–30 min***	Phenytoin: 15 mg/kg IV. 10 mg/kg repeatable after 20 min (velocity not above 50mg/min)	***Phase 1*** ***30–60 min***	Propofol: 2–4 mg/kg in bolus. Infusion 3–10 mg/kg/h and titolazione to maintain burst-suppression.
	Diazepam: 0.5–1mg/kg IV		Valproic acid: 20 mg/kg (velocity: 5 mg/kg/min)		Midazolam: 0.2 mg/kg (dose max 5 mg). Continuous infusion 0.1–0.3 mg/kg/h
	Clonazepam: 1 mg bolus IV (max 0.5 mg/min). If it is necessary it can be repeated once after 5 min		Levetiracetam: 30 mg/kg (velocity: 5 mg/kg/min)		Thiopental: 3–5 mg/kg IV. Loading dose in 20 s. continuous infusion: 1–3 mg/kg/h with the aim to maintain burst suppression
	Fenobarbital: 10 mg/kg (range 10–20) bolus IV. Infusion max dose: 100 mg/min		Lacosamide (>16 years): loading dose 200 mg. Dose max/die 400 mg repeatable once		Pentobarbital: 5–15 mg/kg bolus IV. Continuous infusion to maintain burst suppression (0.5–3 mg/kg/h)
